# Knowledge, attitudes and practices of parents towards antibiotic use in rural communities in Peru: a cross-sectional multicentre study

**DOI:** 10.1186/s12889-022-12855-0

**Published:** 2022-03-07

**Authors:** Jose Luis Paredes, Rafaella Navarro, Takashi Watanabe, Flavia Morán, Maria Pia Balmaceda, Andrea Reateguí, Raul Elias, Miguel Bardellini, Theresa J. Ochoa

**Affiliations:** 1 Instituto de Medicina Tropical Alexander von Humboldt, Universidad Peruana Cayetano Heredia, Av. Honorio Delgado 430, San Martín de Porres, 15102 Lima, Peru; 2 Facultad de Medicina, Universidad Peruana Cayetano Heredia, Lima, Peru; 3 School of Public Health, University of Texas Health Science Center at Houston, Houston, TX USA

**Keywords:** Antibiotic usage, Rural, Knowledge, Parents, Peru

## Abstract

**Background:**

The inappropriate use of antibiotics significantly contributes to the development of antibiotic resistance. There is limited information about the use of antibiotics among parents from rural areas in Peru. This study aimed to describe the knowledge, attitudes and practices towards antibiotics among parents of children < 5 years of age from rural communities in Peru; to explore the association between knowledge and attitudes towards antibiotics and to explore determinants of low knowledge and self-medicating his/her child with antibiotics.

**Methods:**

Cross-sectional study in six rural primary health centres in Peru using a self-administered survey. Crude and adjusted Prevalence Ratios (PR), and 95% Confidence Intervals (95% CI) were calculated to explore determinants of low knowledge and of having self-medicated his/her child with antibiotics. Linear regression was used to explore the association between knowledge and attitudes.

**Results:**

A total of 231 parents were included. The largest gap in knowledge was among 183 parents (79%) who did not know that antibiotics cannot cure viral infections. The largest gap in attitudes was among 185 participants (80%) that did not disagree with “If I want my child to receive antibiotics, I would not be satisfied if the doctor refuses to prescribe them”. More than half of parents (*n* = 120, 52%) reported having self-medicated his/her child with antibiotics. A positive correlation was found between knowledge and attitudes (Coefficient 0.53, 95% CI 0.38–0.68) after adjusting for the age and the education of the parent. Parents who were < 20 years old were more likely to have low knowledge about antibiotics (crude PR 2.39, 95% CI 1.32–4.34) compared to those aged > 40 years.

Parents who had self-medicated his/her child with antibiotics (*n* = 120, 52%) were more likely to have purchased antibiotics without prescription (aPR 2.70, 95% CI 1.74–4.19) and to have received antibiotics after the recommendation of a pharmacist (aPR 1.79, 95% CI 1.13–2.82).

**Conclusions:**

Knowledge about antibiotics among parents from rural settings in Peru is limited and highlights the need for educational interventions. Public health policies to limit the acquisition of antibiotics without prescription should be implemented.

## Background

Antibiotics have saved millions of lives and have changed the history of infectious diseases. However, in recent years, antibiotic resistance has become a major problem for global public health. Antibiotic resistance challenges the treatment of common infectious diseases, increases mortality rates and treatment costs [1]. Each year, around 700, 000 deaths are caused by resistant infections [2].

Antibiotic resistance occurs naturally; however, inadequate antibiotic use, poor patient adherence to antibiotics and insufficient regulation of antibiotics increase its propagation [3]. The main reason for the development of resistance is the inadequate use of antibiotics. Particularly in developing countries where antibiotic use is often not well regulated, enabling self-medication [4]. More than 50% of antibiotics worldwide are purchased from pharmacies or street vendors without a prescription [5].

Due to the high burden of infectious diseases in children [6], they receive many antibiotics. Also, parents often use these drugs inappropiately [7–10], which is influenced by their knowledge, attitudes and practices towards these drugs [10]. Some studies report important gaps in parents’ understanding of antibiotics: some believe that these drugs can cure viral infections [10], that antibiotics should be used every time a child has fever [7] and that shorter courses of antibiotics are healthier for their children [7, 9].

In Peru, an upper-middle-income country, the rates of antibiotic resistance among common bacteria are high, [11] and parental use of antibiotics without prescription for their children seems to be common [7, 12]. Most studies regarding antibiotic resistance and the use of these drugs have been carried out in urbanized areas [7, 12]. Despite the limited knowledge on the use of antibiotics by parents in rural settings, high rates of resistant pathogens among children in rural communities have been reported [13, 14]. This study aims to describe the knowledge, attitudes and practices towards antibiotics in parents of children < 5 years of age from rural communities in Peru; to explore the association between knowledge and attitudes towards antibiotics and to explore factors associated with low knowledge and with self-medicating his/her child with antibiotics.

## Materials and methods

### Study setting and population

The study was carried out in six primary health centres located in rural communities in Peru, which provide care to 579 children aged less than 5 years (Table 1). A sample size of 232 parents was estimated considering an *α* = 0.05, a power of 80% and a percentage of 50% for the outcomes (low knowledge and having self-medicated his/her child with antibiotics). We included parents of children under 5 years of age, who were older than 18 years and who provided informed consent.

**Table 1:** Characteristics of the primary health care centres included in the study

Department	Province/District	Natural Region	Community Type	Number of children under five years	Total Monetary Poverty 95% CI^**a**^	Water Drainage	Distance to a tertiary hospital
Amazonas	Utcubamba/Yamon	Jungle	Native	76	30–47	Yes	48 km
San Martin	Rioja/Yorongo	Jungle	Rural	86	20–42	No	15 km
Pasco	Oxapampa/Villa Rica	Jungle	Native	51	23–37	No	11 km
Lima	Huarochiri/Mariatana	Highlands	Rural	70	23–38	No	130 km
San Martin	Rioja/Nueva Cajamarca	Highlands	Rural	250	20–37	Yes	15 km
Amazonas	Luya/Lamud	Highlands	Rural	61	24–38	Yes	35 km

^a^95% Confidence Interval of Total Monetary Poverty estimated using the Micro-Level Estimation of Poverty and Inequality method of Chris Elbers, Jean O. Lanjouw Peter Lanjouw [15]

*CI* Confidence Interval

### Study design and procedures

We conducted a cross-sectional study between October 2019 and May 2020. We used convenience sampling to select the participants: all parents who attended the health centre and who met the inclusion criteria were invited to participate. After providing written informed consent, each participant completed the self-administered survey. The survey was verbally administered by a research team member to participants who were unable to read.

This survey had 34 multiple-choice questions and 1 open-ended question. It was divided into 3 sections: (1) sociodemographic factors (age, relationship with the child, education status, number of children, age of older and younger child), (2) use of antibiotics (by the parent and by his/her child in the last 12 months) and (3) knowledge (possible answers: yes/no/I don’t know), attitudes (possible answers: agree/disagree) and practices (possible answers: yes/no) towards antibiotics. The section on practices included the open-ended question that inquired about parents’ reasons for self-medicating his/her child with antibiotics.

This paper-based survey on knowledge, attitudes and practices towards antibiotics was validated in two previous studies [9, 16] and previously administered to parents attending urban and peri-urban health centres in Lima, Peru [7]. We included one additional question on the attitudes section regarding parents expectations of receiving antibiotics (If I want my child to receive antibiotics, medical doctors should agree with my decision and prescribed them) and two questions on the practices section: one about the acquisition of antibiotics after the recommendation of a pharmacist (which seems to be common among Peruvian parents) [12] and one on reasons for self-medicating his/her child with antibiotics (as done previously by Yu et. Al) [9].

### Data management and statistical analysis

Participant’s data was entered into a secure Excel database. The analysis was performed using Stata v.16. We calculated percentages for categorical variables and measures of central tendency: median and interquartile ranges (IQR) for continuous variables.

For the categorization of knowledge, we gave one point for each correct answer and zero if the incorrect answer or the “I don’t know” was selected. Knowledge was classified as low (1–3 points), moderate (4–6 points) or high (≥7 points) [7, 16]. For the attitudes, we gave one point for each desired attitude and zero if the desired attitude was not selected. We categorized attitudes as adequate if participants scored above the median and inadequate if they scored below the median. For the categorization of attitudes, we excluded two questions that addressed participants perception of the physicians’ attitudes towards antibiotics.

To explore the association between low knowledge and inadequate attitudes towards antibiotics we adjusted for parent’s education status. The factors included as potential determinants of low knowledge about antibiotics were: gender [17], paternal age [10], education [7], number of children [18], oldest child age [7] and parental and children’s use of antibiotics in the last 12 months [7]. For the analysis of potential determinants of having self-medicated his/her child with antibiotics, participants were categorized based on their response to the question inquiring in this topic in the section on practices. We explored the association of factors that have been reported to be determinants of self-medication with antibiotics in low and middle-income countries and among Peruvian parents (gender, education, number of children, age of the oldest child, previous use of antibiotics, knowledge about antibiotics, attitudes towards antibiotics, purchasing antibiotics without physician prescription and antibiotics storage at home) [7, 19]. Additionally, we included having received antibiotics after the recommendation of a pharmacist as a potential determinant of having self-medicated his/her child with antibiotics.

Poisson regression with robust variance was used to calculate Prevalence Ratios (PR), 95% Confidence intervals (95% CI) and *p* values in the bivariate and multivariate analysis to study the factors associated with low knowledge and of having self-medicated his/her child with antibiotics. Variables with a *p*-value < 0.2 in the bivariate analysis were included in the multivariate analysis and backwards elimination was used based on the likelihood ratio test.

Bivariate linear regression was used to explore if the knowledge and attitudes scores were associated. A multivariate linear regression was done to adjust the association between knowledge and attitudes for *a priori* defined confounders (the age and the education of the parents).

## Results

### Study population and participant’s characteristics

A total of 259 parents were invited to participate, 28 (10.8%) declined participation and 231 (89.2%) agreed and provided informed consent. About 90 % of the 231 parents (*n* = 207, 89.6%) were mothers and the median age was 30 years (IQR 24–35). A total of 82 (35.5%) participants had one child, 72 (31.2%) had two and 77 (33.3%) had three or more children. The median age of the oldest child was 7 years (IQR 2.5–13 years) and the median age of the youngest child was 2 years (IQR 0–4 years). Regarding parent’s education, 6 (2.6%) reported no education, 83 (35.9%) had primary education, 116 (50.2%) had secondary education and 26 (11.3%) had tertiary education. Ten participants (4.3%) did not know how to read.

In the 12 months before the study, 143 parents (61.9%) had used antibiotics, 76 (32.9%) had not used antibiotics and 12 (5.2%) did not remember. A total of 147 children (63.6%) had used antibiotics within the 12 months previous to the study, 53 children (22.9%) had used antibiotics previous to the last 12 months, 16 (6.9%) had never received antibiotics and the parents of 15 children (6.5%), did not remember if their children had received antibiotics in the past.

### Knowledge, attitudes and practices about antibiotics

Of the 231 participants, 79 (34.2%) had low knowledge about antibiotics, 115 (49.8%) had moderate knowledge and 37 (16.0%) had high knowledge. Four participants (1.7%) replied correctly to all the questions in the knowledge section and thirteen participants (5.6%) answered only one question correctly. The largest gap in antibiotic knowledge was among 183 participants (79.2%) who did not know that antibiotics cannot cure viral infections (Table 2).

**Table 2:** Knowledge, attitudes, and practices towards antibiotics of parents from children under 5 years in primary health care centres from rural communities in Peru (*N* = 231)

Question (Correct/desired answer)	Correct/Desired responses, N (%)
**Knowledge**	
Antibiotics can treat bacterial infections. (Yes)	164 (71.0)
Antibiotics can cure viral infections. (No)	48 (20.8)
Antibiotics must be taken once a child has a cold. (No)	87 (37.7)
Antibiotics are the same as medications used to relieve pain and fever such as acetaminophen. (No)	96 (41.6)
Penicillin is an antibiotic. (Yes)	123 (53.2)
Children can be allergic to antibiotics. (Yes)	204 (88.3)
The effectiveness of treatment is reduced if a full course of antibiotics is not completed. (Yes)	126 (54.6)
Taking fewer antibiotics than prescribed is healthier than taking the full course prescribed. (No)	99 (42.9)
Is the efficacy better if the antibiotics are newer and more costly? (No)	80 (34.6)
**Attitudes**	
Leftover antibiotics are good to keep at home in case I might need them for my child later on. (Disagree)	137 (59.3)
It is good to be able to get antibiotics for my child from siblings, relatives, or friends without having to see a doctor. (Disagree)	180 (77.9)
It is good to be able to buy antibiotics over the counter at the pharmacy. (Disagree)	116 (50.2)
It is appropriate to use antibiotics when my child has a sore throat because otherwise, he or she might catch something more serious. (Disagree)	69 (29.9)
Antibiotics speed up recovery from a cold. (Disagree)	65 (28.1)
I usually stop giving antibiotics to my child when he or she starts feeling better. (Disagree)	103 (44.8)
I will stop giving my child an antibiotic if he or she has a skin reaction or gets side effects. (Agree)	204 (88.3)
I usually will look at the expiry date of antibiotics before giving them to my child. (Agree)	214 (92.6)
Doctors often take time to consider carefully whether my child needs to be prescribed antibiotics or not. (Agree)	181 (78.4)
Doctors often take time to inform parents how antibiotics should be used for their children. (Agree)	192 (83.1)
Antibiotics should be administered in all cases once a child has a fever. (Disagree)	73 (31.6)
If I want my child to receive antibiotics, I would not be satisfied if the doctor refuses to give them. (Disagree)	63 (27.3)
**Practices**	
Has purchased antibiotics without physicians’ prescription. (No)	95 (41.1)
Sometimes, often, or always stores antibiotics at home. (No)	90 (39.0)
Have self-medicated their children with antibiotics. (No)	111 (48.1)
Believes it is reasonable to not visit a doctor if their child’s condition is not very serious. (No)	86 (37.2)
Obtaining antibiotics without a prescription is a reason for self-medication. (Yes)	64 (27.7)
Has received antibiotics after pharmacist advice. (No)^a^	76 (33.0)

^a^missing value on 1 participant

The median score obtained by the 231 participants in the attitudes section was 5 (IQR 4–7); 91 participants (39.4%) had adequate attitudes towards antibiotics and 140 (60.6%) had inadequate attitudes towards antibiotics. The largest gap was among the 185 participants (80.1%) that did not disagree with “If I want my child to receive antibiotics, I would not be satisfied if the doctor refuses to prescribe them” and among 166/230 parents (72.2%) who did not disagree with “Antibiotics speed up the recovery from a cold” (Table 2).

In the practices section, the largest gaps were among the 167 (72.3%) parents who stated that obtaining antibiotics without a prescription is not a reason for self-medication and among the 155 (67.1%) parents who received antibiotics after the recommendation of a pharmacist without having a prescription. More than half of parents (51.9% *n* = 120) reported having self-medicated his/her child with antibiotics (Table 2). The most common reasons reported for this practice were: “I had some antibiotics left at home that were previously prescribed for a similar condition” among 59 (25.5%) parents, “I don’t consider the need to visit the doctor because of mild illness of my child” among 45 (19.5%) parents, “I had not enough money to go to the health centre” in 39 (16.9%) parents, “I did not have time to go to the paediatrician” in 10 (4.3%) parents and “I had not enough time to go to the health centre” among 3 (1.3%) parents.

### Association between knowledge and attitudes towards antibiotics

There was strong evidence (*p* < 0.001) of a positive linear association between the knowledge and the attitudes scores. For each increase in the score on knowledge, the score on attitudes increased 0.59 points (Coefficient 0.59, 95% CI 0.46–0.72). After adjusting for the age and the education of the parent, the score on attitudes increased 0.53 points (Coefficient 0.53, 95% CI 0.38–0.68) per increase in the score on knowledge. The evidence for this association remained strong (*p* < 0.001) in the multivariate analysis.

### Factors associated with low knowledge about antibiotics

Regarding factors associated with low knowledge about antibiotics (Table 3), 56.5% (13/23) of those aged ≤20 years had low knowledge about antibiotics compared to 23.6% (13/55) of those aged > 40 years. Participants who were ≤ 20 years old had a 2-times higher prevalence of low knowledge (crude PR 2.39, 95% CI 1.32–4.34) compared to participants aged > 40 years. No other factor was associated with low knowledge about antibiotics.

**Table 3:** Determinants of low knowledge about antibiotics in parents from primary health care centres in rural communities in Peru (*N* = 231)

Characteristic	Moderate/high knowledge	Low knowledge	Crude PR (95% CI)	***P*** value^**1**^
Gender				
Male	16 (66.7)	8 (33.3)	1	0.925
Female	136 (65.7)	71 (34.3)	1.03 (0.57–1.87)	
Age of respondent				
≤ 20	10 (43.5)	13 (56.5)	2.39 (1.32–4.34)	0.0525
21–30	52 (65.8)	27 (34.2)	1.44 (0.82–2.55)	
31–40	48 (64.9)	26 (35.1)	1.49 (0.84–2.62)	
> 40	42 (76.4)	13 (23.6)	1	
Education				
None or primary	54 (60.7)	35 (39.3)	1.27 (0.89–1.81)	0.191
Secondary or tertiary	98 (69.0)	44 (31.0)	1	
Number of children				
One or two	102 (66.2)	52 (33.8)	1	0.844
Three or more	50 (64.9)	27 (35.1)	1.04 (0.71–1.51)	
Age of her/his older child				
1–2	55 (59.8)	37 (40.2)	1	0.115
> 3	97 (69.8)	42 (30.2)	0.8 (0.5–1.1)1	

*PR* Odds ratio, *CI* Confidence interval

^*^adjusted for the other variables included in the model

^1^*p* value derived from the Likelihood ratio test in the bivariable analysis

### Factors associates with having self-medicated his/her child with antibiotics

Regarding factors associated with having self-medicated his/her child with antibiotics (Table 4), 74% (100/136) of parents who had purchased antibiotics without prescription had self-medicated his/her child with antibiotics compared to 21% (20/95) of parents who had not previously purchased antibiotics without prescription. Furthermore, 67% (103/154) of parents who had received antibiotics after the recommendation of a pharmacist had self-medicated his/her child with antibiotics compared to 22% (17/76) of parents who had not received antibiotics after the recommendation of a pharmacist. Having self-medicated his/her child with antibiotics was more common among parents who purchased antibiotics without prescription (adjusted PR 2.70, 95% CI 1.74–4.19) and among those who received antibiotics after the recommendation of a pharmacist (adjusted PR 1.79, 95% CI 1.13–2.82).

**Table 4:** Determinants of having self-medicated his/her child with antibiotics in parents from primary health care centres in rural communities in Peru (*N* = 231)

Characteristic	Has not self-medicated his/her child with antibiotics	Has self-medicated his/her child with antibiotics	Crude PR (95% CI)	***P*** value^**1**^	Adjusted PR (95% CI)* (***n*** = 230)	***P*** value^**2**^
Knowledge about antibiotics						
Medium/high	74 (48.7)	78 (51.3)	1	0.789	–	–
Low	37 (46.8)	42 (53.2)	1.03 (0.80–1.34)		–	
Attitudes						
Adequate	49 (53.9)	42 (46.2)	1	0.167	–	–
Inadequate	62 (44.3)	78 (55.7)	1.21 (0.92–1.58)		–	
Sex						
Male	16 (66.7)	8 (33.3)	1	0.188	–	–
Female	136 (65.7)	71 (34.3)	1.43 (0.84–2.44)		–	
Age of respondent						
≤ 20	8 (34.8)	15 (65.2)	1	0.605	–	–
21–30	39 (49.4)	40 (50.63)	0.78 (0.54–1.12)		–	
31–40	37 (50.0)	37 (50.0)	0.77 (0.53–1.12)			
> 40	27 (49.1)	28 (50.9)	0.78 (0.53–1.16)			
Education						
No education / primary	48 (53.9)	41 (46.1)	1.27 (0.89–1.81)	0.191	–	–
Secondary/tertiary	63 (44.4)	79 (55.6)	1		–	
Number of children						
One or two	102 (66.2)	52 (33.8)	1	0.782		–
Three or more	50 (64.9)	27 (35.1)	0.96 (0.74–1.26)		–	
Has purchased antibiotics without physicians’ prescription						
No	75 (78.9)	20 (21.1)	1	< 0.01	1	< 0.01
Yes	36 (35.5)	100 (73.5)	3.49 (2.33–5.23)		2.70 (1.74–4.19)	
Sometimes, often, or always stores antibiotics at home						
No	60 (66.7)	30 (33.3)	1	< 0.01	–	–
Yes	51 (36.2)	90 (63.8)	1.9 (1.4–2.6)		–	
Has received antibiotics after the recommendation of a pharmacist^3^						
No	59 (77.6)	17 (22.4)	1	< 0.001	1	0.012
Yes	51 (33.1)	103 (66.9)	3.0 (1.9–4.6)		1.79 (1.13–2.82)	

*CI* Confidence interval, *PR* Prevalence ratio, *aPR* Adjusted prevalence ratio

^*^adjusted for the other variables included in the model

^1^*p* value derived from the Likelihood ratio test from the bivariable analysis

^2^*p* value derived from the Likelihood ratio test from the multivariable analysis

^3^missing value on 1 participant (*n* = 230)

## Discussion

Our findings suggest that one-third of parents of children below 5 years of age attending primary health centres in rural communities in Peru have low knowledge and inadequate attitudes towards antibiotics. We found important gaps in the knowledge and attitudes of parents, such as the 79% who did not know that antibiotics cannot cure viral infections and 80% that did not disagree with “If I want my child to receive antibiotics, I would not be satisfied if the doctor refuses to prescribe them”. More than half (52%) reported having self-medicated his/her child with antibiotics and 69% had received antibiotics after the recommendation of a pharmacist. Knowledge and attitudes had a positive linear correlation. Low knowledge was more common among parents aged ≤20 years compared to those aged > 40 years. Having self-medicated his/her child with antibiotics was associated with having purchased antibiotics without prescription and having received antibiotics after the recommendation of a pharmacist.

Most parents believed that antibiotics cure viral infections and that antibiotics speed up the recovery from a cold, highlighting their low awareness about the inefficacy of antibiotics against common colds and viral infections, which seems to be common among parents worldwide [10]. Low parental knowledge about antibiotics influences parents' expectation of receiving antibiotics for upper respiratory tract infections [10]. Upper airway infections are the most common cause of outpatients clinic consultation in Peru [6], especially among children from rural populations [20].

In our study, most parents reported that if they want their child to receive antibiotics, medical doctors should agree with their decision and prescribe them which is similar to the findings of another study among 218 physicians in Lima, where higher satisfaction of the mothers with antibiotic prescription was associated with inadequate use of antibiotics by physicians [8]. This phenomenon has also been recognized in other settings. Among parents in the UK, the expectations for antibiotics increased the physicians’ intentions to prescribe them, without increasing the probability of a bacterial infection [21]. In the United states, one study that explored multiple factors potentially associated with inappropriate antimicrobial prescribing found that physicians’ perceptions of parental expectations for antimicrobials was the only predictor [22].

The “National Plan against antimicrobial resistance” was created in Perú in 2017 to sensitize the general population about antimicrobial resistance by publishing articles about this topic on social media and in the national press [23]. This strategy takes for granted access to social media and to the national press, which might be limited in rural settings. The gaps in the knowledge and attitudes reported in this study may be used for the development of informative materials that can address these topics in rural settings and improve parents' understanding of antibiotics.

In our study, almost 60% of the parents reported having purchased antibiotics without prescription and almost 70% had received antibiotics after the recommendation of a pharmacist. In the Americas, only 9% of countries have a national antimicrobial resistance plan and antimicrobial medicines are available without prescription in 51% of countries in the region [24]. Despite the prohibition of the purchase of antibiotics without prescription in 1962 in Peru [25], it is possible to obtain them over-the-counter. One study in Lima revealed that 79% of pharmacies sold antibiotics without prescription [26, 27]. The implementation of strategies to adequately limit the acquisition of antibiotics without prescription should be a priority in Peru, especially focusing on monitoring and controlling the establishments where antimicrobials are sold [26].

The use of antibiotics without prescription in children by their parents has also been recognized in previous studies in Peru with different frequencies: 13% in parents at drug stores in Lima [12] and 29% in parents from an underprivileged area in Iquitos (rainforest) [14]. We found that 52% of parents had self-medicated his/her child with antibiotics, which implies a higher use of antibiotics without prescription by parents in rural populations and has been described in other settings [9]. In our study, the factors associated with having self-medicated his/her child with antibiotics were purchasing antibiotics without physicians’ prescription and having received antibiotics after the recommendation of a pharmacist. Both factors can be controlled by the implementation of public health policies that limit the acquisition of antibiotics without prescription.

The fact that knowledge and attitudes were not related to the use of antibiotics without prescription in their children gives rise to the question “If this practice is not a matter of knowledge or attitudes, why are parents in rural settings in Peru self-medicating their children with antibiotics?”. In our study, 16.8 and 4.3% of parents recognized not having enough money or time to go to the health centres as reasons for self-medicating their children with antibiotics respectively, which means that more than 20% experience inadequate access to health centres. This aspect is similar to the findings of a study in Lima, where 21% of parents reported getting antibiotics without prescription because medical attention takes too long and 18.4% reported no access to a health centre at the time they needed it [12]. Both studies highlight that access to healthcare centres is fundamental to limit self-medication with antibiotics in Peruvian parents. Strengthening access to healthcare seems to be important for addressing inadequate antibiotic use and limiting the development of antimicrobial resistance. We recommend that further research should aim to understand the relationship between access to health care and self-medication with antibiotics. Access to health care is a human right, however, in some settings, it is not guaranteed for all equally and low access to health centres in rural areas has been previously reported in other settings [28]. Improving accessibility to health care, might be fundamental to decrease the use of antibiotics without prescription in rural settings.

Given the low knowledge and high rates of self-medication reported in our study, we believe that it is fundamental to develop strategies to address knowledge and self-medication with antibiotics in parents from rural communities in Perú. One review of supply-side interventions to improve practices of antibiotic prescribers in low- and middle-income countries reported that of 40 interventions, 20 had a positive result regarding antibiotic prescribing; most of them using mixed methods interventions [29]. The World Health Organization recommends that all health centres should have training on Antimicrobial Resistance [30] and it is recommended that a multidisciplinary team should address this topic. Even though this plan is focused on tertiary hospitals, the actions to control antibiotic resistance in primary health centres should not be neglected. Furthermore, many rural health centres have limited personnel and scarce resources, therefore designating professionals for specific roles in this context may not be possible. Therefore, we believe that the use of educational materials (such as brochures and posters) at the health centres about the dangers of self-medication with antibiotics and the adequate use of these drugs and the implementation of campaigns regarding antibiotic resistance, could be feasible ways to educate parents in rural primary health centres.

The results of this study must be interpreted considering its limitations. First, parents who agreed to participate were probably those who felt more comfortable replying to a knowledge survey and those with higher education. However, in our study, 11.3% of parents had tertiary education, which is similar to the national estimates for rural communities reported in the last census (9.3%) [15]. Also, we decided to invite parents who could not read (5.9% of parents in our study), in whom the survey was applied verbally by the physician. Second, we only enrolled parents at health centres who might have more access to health care and could be more conscious of their children’s health. This might imply that our findings could not represent all parents from rural communities in Peru, but only those with access to health care. However, the gaps in knowledge and attitudes, and the high percentage of self-medication with antibiotics found in our study should warrant further research to identify these aspects in parents who do not attend primary health centres. Furthermore, given the limited number of participants per location/region, differences in the knowledge attitudes and practices towards antibiotics were not explored, which could help in the development of targeted interventions in specific regions. Finally, recall bias may have influenced the results, as data collection was performed using self-administered surveys based on events or experiences regarding antibiotic use that may have taken place in the past and parents might have intentionally omitted incorrect practices, which might have underestimated certain aspects on antibiotic use. The most important strength of this study is that, to our knowledge, this is the first multicenter study addressing knowledge, attitudes, and practices in parents from rural populations in Peru and our findings can give rise to multiple hypotheses and interventions in this vulnerable population.

## Conclusions

This study highlights important gaps in the knowledge and attitudes of parents in rural communities in Peru regarding the use of antibiotics. Our findings underline the need to implement interventions to increase the knowledge of Peruvian parents from rural communities on antibiotic use and may help in the development of educational campaigns in this population. Furthermore, we found alarming rates of having self-medicated his/her child with antibiotics and of obtaining antibiotics after the recommendation of a pharmacist. It is fundamental to develop public health strategies to limit the acquisition of antibiotics without prescription, especially in rural areas in Peru.

## Data Availability

The datasets used and/or analysed during the current study are available from the corresponding author on reasonable request.

## Abbreviations

IQR: Interquartile range
PR: Prevalence ratio
aPR: Adjusted Prevalence Ratio
95% CI: 95% Confidence interval
